# Supplementary figures and images for: A multistage sequencing strategy pinpoints novel candidate alleles for Emery-Dreifuss muscular dystrophy and supports gene misregulation as its pathomechanism
Source: eBioMedicine. 2019 Dec 17;51:102587. doi: 10.1016/j.ebiom.2019.11.048 (PMC7000448; doi:10.1016/j.ebiom.2019.11.048)

**Supplementary Material**

Figure S1: Effects of tested PLPP7 and TMEM38a mutations on nuclear shape


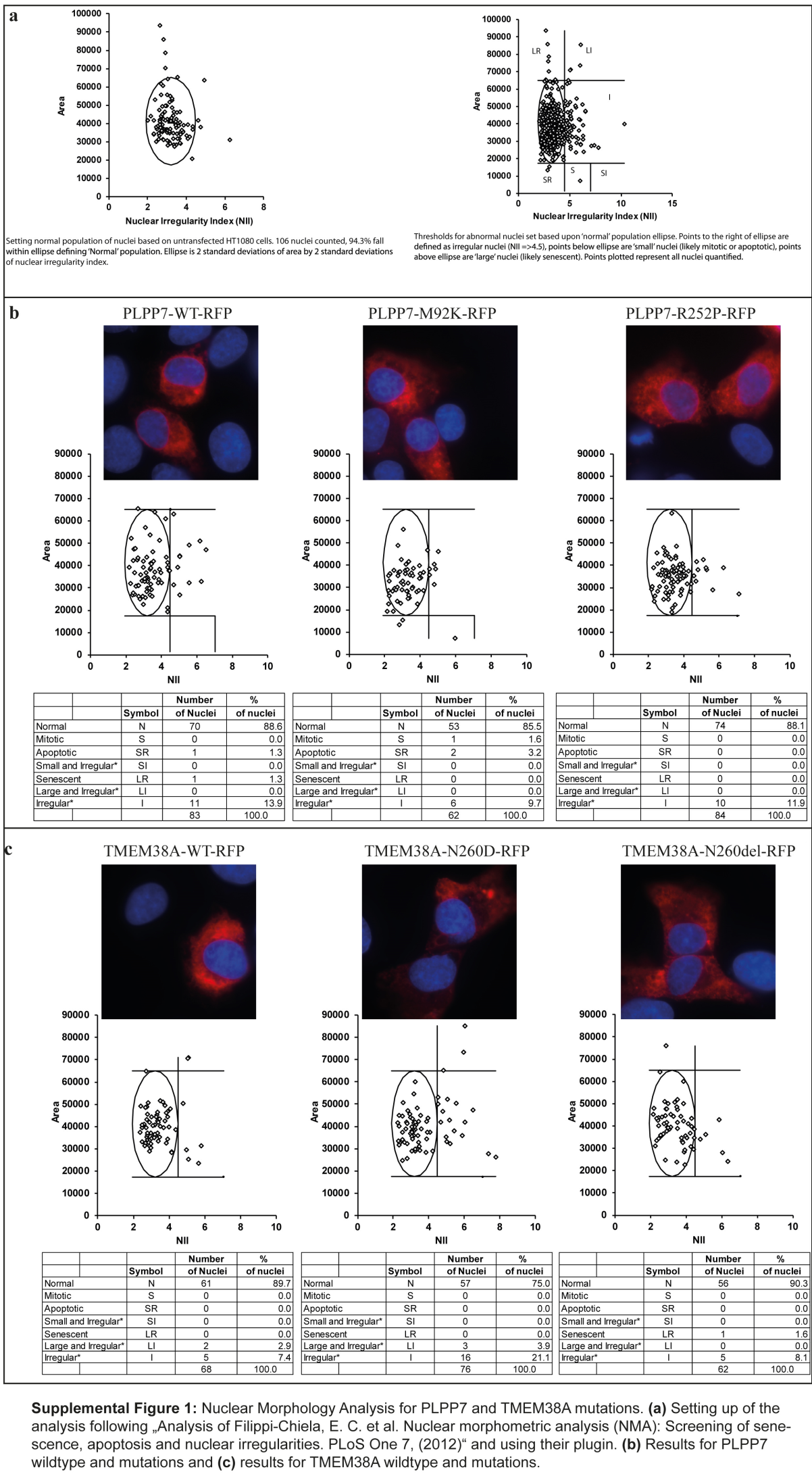

Supplement: Supplementary file 6 [file mmc6.docx]
